# The Medical Profession

**Published:** 1901-09-07

**Authors:** 


### The Hospital

A Journal of

The Medical Sciences and Hospital Administration.

Vol. XXX.—No. 780.] Edited by Sir Henry Burdett, K.C.B., and Solomon C. Smith, M.D., M.R.C.P. [September 7, 1901.

### The Medical Profession.

The approach of October 1st, the day on which a large number of young men will find themselves definitely committed to the pursuit of a medical career, is a period which cannot to many persons and in many families be wholly free from anxiety with regard to the wisdom of the choice. The circumstances by which this choice has been determined may be almost infinitely various, and may range over the whole fields of opportunity, of fitness, and of inclination. The latter may not infrequently be directed towards medicine by observation of the important part played by the doctor in many households, and by recognition of the manner in which his presence removes anxiety or in which his skill assuages pain. "There are men, and classes of men," wrote Robert Stevenson, "that stand above the common: the soldier, the sailor, and the shepherd not unfrequently; the artist rarely; rarelier still the clergyman; the physician almost as a rule. He is the flower (such as it is) of our civilisation; and when that stage of man is done with, and only remembered to be marvelled at in history, he will be thought to have shared as little as any in the defects of the period and most notably exhibited the virtues of the race. Generosity he has, such as is possible to those who practise an art, never to those who drive a trade; discretion, tested by a hundred secrets; tact, tried in a thousand embarrassments; and, what are more important, Herculean cheerfulness and courage. So it is that he brings air and cheer into the sick room, and often enough, though not so often as he wishes, brings healing." In the presence of such appreciation as this, and it is felt, and in their own language uttered, by hundreds who have no power of finished expression, it is natural that the impulses of a generous boy should impel him towards a life in which he, too, may come to be looked upon with like regard, and to be expected with like eagerness. Few boys, again, at least among those who are on the way to become Englishmen, could read such a book as "Two Years Ago," without wishing to emulate the exploits of its hero; and turning from fiction to fact, which is often stranger than fiction, it may truly be said that in an Empire on which the sun never sets, there is no place and no day in which members of the medical profession may not be found at the post of duty, wherever this may be, in the palaces of the wealthy, in the slums of cities, in the lonely hamlet, in the beleaguered garrison, in the tented field. No other calling affords equal opportunities of observing and of studying the essentials, as distinguished from the accidentals, of human nature; no other so entwines the love of truth with every fibre of the intellectual organism; no other affords even approximately equal opportunities of doing good.

These, no doubt, are weighty considerations; but there are yet others which must be taken into account, by every young man or by his friends, in determining the choice of his profession. There must be reasonable ground for expecting, from that which is chosen, that it will afford an honest living, in the social position of a gentleman. In order to arrive at some trustworthy opinion with regard to the advantages offered by medicine in this respect, the late Sir James Paget, assisted by two of his colleagues, traced out, more than 30 years ago, the careers of 1,000 medical students, who had entered at St. Bartholomew's Hospital, and had left it 15 years before the inquiry commenced. His figures would occupy more space than it is possible for us to bestow upon them; but, if we consider that it is a necessary condition of success, in any calling, that it should be followed with due diligence and for a sufficient time, and if we leave out of account those among the 1,000 students who early turned away to other occupations, and those who died either before, or shortly after, they became qualified as practitioners, the number of those who persevered will be reduced to 776, out of whom only 56 are recorded to have failed entirely. One hundred and twenty-four are said to have had a "very limited" success; and the remaining 596, or more than 75 per cent. of the whole, obtained respectable positions and comfortable maintenance, increased, in the case of 66, to "considerable," and, in the case of 23, to "distinguished" success. With regard to the determining causes of these results, Sir James declared that nothing appeared to him to be more certain than that the personal character, the very nature, the will, of each student had far greater force in determining his career than any helps or hindrances whatever. "All my recollections," he continued, "lead me to believe that every student may draw from his daily life a very likely forecast of his life in practice, for it will depend on himself a hundredfold more than on circumstances. The time and the place, the work to be done and its responsibilities, will change, but the man will be the same, except in so far as he may change himself." In pursuance of the same idea, it may be pointed out that not the least of the advantages attaching to medicine as a pursuit is the variety of work which it affords, and the opportunities thus given to men of different tastes and qualifications. There seldom need be a question of putting a square peg into a round hole. The holes are not only numerous, but they are of all conceivable shapes and sizes; and the young doctor who cannot find one to fit him must be either exceptionally angular or exceptionally unyielding, or both. For any who are thus unfortunate, it would perhaps be difficult to discover an occupation entirely suited to their requirements.

